# *Lactobacillus johnsonii* 6084 alleviated sepsis‐induced organ injury by modulating gut microbiota

**DOI:** 10.1002/fsn3.2989

**Published:** 2022-07-22

**Authors:** Shichao Han, Haotian Zheng, Fu Han, Xiaowei Zhang, Geng Zhang, Shuaijun Ma, Kepu Liu, Weijun Qin, Gaofeng Wu

**Affiliations:** ^1^ Department of Urology, Xijing Hospital Fourth Military Medical University Xi'an China; ^2^ BGI Education Center University of Chinese Academy of Sciences Shenzhen China; ^3^ Department of Burns and Cutaneous Surgery, Xijing Hospital Fourth Military Medical University Xi'an China; ^4^ Department of Obstetrics and Gynecology Peking University Shenzhen Hospital Shenzhen China

**Keywords:** inflammation, *Lactobacillus johnsonii* 6084, microbiota, sepsis

## Abstract

Sepsis is a public cause of death in intensive care unit patients. Probiotics were widely used to increase the survival rate of sepsis by a series of clinical research. The purpose of this research was to investigate the therapeutic effects of *Lactobacillus johnsonii* 6084 in septic mice. Sepsis mouse model was induced by LPS treatment. The influence of *L. johnsonii* 6084 on the protection of organ injury induced by sepsis was explored. Moreover, the composition of gut microbiota was studied to clarify the mechanism of *L. johnsonii* 6084 therapeutic effect on sepsis. *L. johnsonii* 6084 treatment could conspicuously decrease the mortality and organ injury of sepsis. The reduction of gut microbial diversity and richness in septic mice were moderated by the administration of 6084. The abundance of *Bacteroidetes* and *Proteobacteria* were change by LPS treatment while restored by *L. johnsonii* 6084. To conclude, probiotic 6084 may has optimistic result on reducing mortality of sepsis through rebalancing gut microbiota.

## Graphical abstract

Probiotic Lactobacillus johnsonii 6084 has a positive effect on reducing mortality of sepsis through rebalancing gut microbiota.

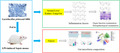

## 1 INTRODUCTION

Sepsis, defined as life‐threatening organ dysfunction caused by a dysregulated host response to infection (Fernando et al., 2018), is a general inflammatory illness with complex biological responses of body to various noxious stimuli, such as bacteria, virus which affects 5 million patients dead worldwide each year (Cecconi et al., 2018; Fleischmann et al., 2016; Kadri et al., 2017), which account for a main cause of death in intensive care units (ICU) worldwide (Rello et al., 2017). Although some therapies such as anti‐infection, organ protection, and fluid resuscitation were used to ameliorate the symptoms of sepsis; however, the effective treatment strategies still have not been developed (Ferrer et al., 2014; Gu et al., 2015; Russell et al., 2017). Therefore, it is urgent to access new insights of the treatment for sepsis.

During sepsis, the composition of intestinal flora is severely distorted, with a loss of symbiotic bacteria and overgrowth of potentially pathogenic microorganisms (Kullberg et al., 2021). However, the gut microbiota lives in the digestive tracts of hosts, which is the body's first line to resistance against the invasion of external pathogens (Glenwright et al., 2017). The self‐colonized intestinal microbial community is not only closely related to the digestion and absorption of nutrients but also plays a very important role in the regulation of the host's immune response (Han et al., 2020). Intestinal microbial disorders are closely related to many diseases, such as ulcerative colitis, inflammatory bowel disease (IBD), autoimmune disease, and sepsis (Paramsothy et al., 2017). Several studies have shown that sepsis could result in intestinal microbial disorders, which in turn exacerbate the development of sepsis (Adelman et al., 2020; Zaborin et al., 2014). The immunomodulatory properties of the gut microbiome provided a striking prospect for sepsis prevention and treatment. It is reported that a well‐adjusted gut microbiota has a protecting role for inflammatory disease (Haak et al., 2018; Han et al., 2020). Thus, we hypothesized if restoring the balance of gut microbiota could have a protective effect on sepsis. There are many ways to restore disturbed gut microbes while probiotics are widely used.

Probiotics are live microbes that can have a valuable effect on hosts (Morelli & Capurso, 2012). Probiotics usually colonize at the intestinal tract of human or animal in a relatively stable amount which could interact with the host cells or intestinal microbiota to benefit human health, such as regulating the body's immune response (Lahner et al., 2016; Zou et al., 2020). It is reported that supplementation of probiotics could alleviate various inflammatory diseases by reconstructing the composition of intestinal microbiota (Tsui et al., 2021; Wang et al., 2021). Previous studies from our group have shown that oral supplementation of mice with probiotics significantly reduced inflammation induced by LPS (Han et al., 2020). *Lactobacillus johnsonii*, is one of the many microbes that exist in in the human intestine, is thought to be beneficial to human general health and well‐being (Klaenhammer et al., 2007; Zhang et al., 2021). However, little is known of the valuable effects of *L. johnsonii* in improving sepsis. We hypothesized that *L. johnsonii* could protect organ injury caused by sepsis by regulating gut microbiota.

In the current study, we studied the effect of *L. johnsonii*, a main ingredient of yogurt, on septic mice, and explored the underlying mechanism. The expression levels of inflammatory factors and constitution of gut microbiota were examined to investigate the effect of *L. johnsonii* 6084. The results herein discussed suggest that *L. johnsonii* 6084 can protect visceral organs caused by sepsis through restoring gut microbiota. As such, diets supplemented with probiotics could alleviate organ damage caused by sepsis.

## 2 MATERIALS AND METHODS

### 2.1 Animals

Healthy male BALB/c mice used in this study (6–8 weeks old, average weight 20 g) were obtained from the Experimental Animal Center of Air Force Medical University. The animal experiments protocols in this study followed the institutional guidelines agreed by the Ethics Committee of the Air Force Medical University.

### 2.2 Bacteria and media

*L. johnsonii* 6048 were bought from China Center of Industrial Culture Collection. *L. johnsonii* 6084 were cultured in MRS medium growth medium supplemented with 1% lactose. Then, 100 μl of *L. johnsonii* 6084 was added to 5‐ml basal medium incubated at 180 rpm, at 37°C for 12 h which the final OD was 1. The CFU of *L. johnsonii* 6084 was determined by MRS agar medium.

### 2.3 Animal model and *L. johnsonii* treatment

Septic mouse model was established through intraperitoneally injected with 1 mg/kg LPS (Sigma‐Aldrich) at the beginning of experiment, with a second dose administered 4 days after the first injection. Mice were randomized into four groups (*n* = 8 per group): Control group (mice were intragastrically administered only with 300 μl/day PBS for 1 week); LPS group (mice were administered with PBS after LPS administration); LPS + *L. johnsonii* 6084 group (mice were intragastrically administered with *L. johnsonii* 6084 for 1 week after LPS administration); *L. johnsonii* 6084 group (mice were intragastrically administered with *L. johnsonii* 6084 for 1 week without LPS administration). Mice were intragastrically administered with 300 μl/day *L. johnsonii* 6084 (1 × 10^9^ CFU/ml) or 300 μl/day PBS once every other day. The concentration of LPS increased to 15 mg/kg when monitor the survival rate (*n* = 10 per group). Mice were anesthetized with isoflurane after treatment 3 days (the total treatment time was 1 week). Blood in mice was obtained by cardiac blood collection under anesthesia. Then, the lung, small intestine, liver, and kidney tissues were collected and divided into two parts. Feces were obtained from cecum and stored at liquid nitrogen for gut microbiota analysis.

### 2.4 Weight and sampling

Body weight of mice was recorded for all groups. The serum and tissues were collected after LPS treatment 1 week. Fresh colon samples from all groups were collected and immediately frozen using liquid nitrogen for further Microbial DNA extraction and Illumina MiSeq sequencing.

### 2.5 Enzyme‐linked immunosorbent assay (elisa)

Blood was collected from the left ventricle of all group mice. The concentrations of Interleukin‐1β (IL‐1β) and tumor necrosis factor α (TNF‐α) in serums were examined using elisa kits (Jiancheng).

### 2.6 Hematoxylin and Eosin (H&E) Staining

Tissues (Liver, Kidney, Lung, and Gut) were fixed in 4% paraformaldehyde, dehydrated in alcohol, and embedded in paraffin. The samples were cut into 4‐μm thick sections and deparaffinized and stained with hematoxylin and eosin (H&E) for histological analysis.

### 2.7 RNA isolation and quantitative real‐time PCR

Total RNA from tissues was extracted using TRIzol methods (Invitrogen) and reversely transcribed into cDNA through PrimeScript™ RT Kit (Takara). SYBR® PremixEx Taq™ II and Bio‐Rad CFX system were used to analyze the expression of genes. Quantitative RT‐PCR data were determined based on cycle threshold (Ct) and normalized to internal loading control genes GAPDH. The primers sequences are listed in Table 1.

**TABLE 1:** The sequences of primers used for RT‐PCR

Names	Species	Sense	Antisense
IL‐1β	Mouse	CAACCAACAAGTGATATTCTCCATG	GATCCACACTCTCCAGCTGCA
TNF‐α	Mouse	TATGGCCCAGACCCTCACA	GGAGTAGACAAGGTACAACCCATC
IL‐6	Mouse	CAACGATGATGCACTTGCAGA	CTCCAGGTAGCTATGGTACTCCAGA
MCP‐1	Mouse	AGCAGCAGGTGTCCCAAAGA	GTGCTGAAGACCTTAGGGCAGA
GAPDH	Mouse	TGTGTCCGTCGTGGATCTGA	TTGCTGTTGAAGTCGCAGGAG

### 2.8 DNA extraction and MiSeq sequencing

E.Z.N.A.® Stool DNA Kit was used to extract bacterial DNA (Omega BioTek) following the manufacturer's instructions. The DNA samples were assessed by PCR with the primer (27F/1492R) targeting the 16S rRNA gene. The purity and quality of the DNA samples were examined using 1% agarose gels containing ethidium bromide and then the DNA samples were sent for MiSeq sequencing.

MiSeq sequencing was used to analysis the structure of gut microbiota (Genergy Biotech, Shanghai, China) targeting the V3–V4 region of the bacterial 16S rRNA gene using primers 341F (5’–CCTACGGGNGGCWGCAG–3’) and 785R (5’–GACTACHVGGGTATCTAATCC–3’). We used the Sequence Read Archive (SRA) database to deposit raw reads. Operational taxonomic units (OTUs) were clustered with a 97% similarity cutoff using UPARSE. UCHIME was used to identify and remove chimeric sequences. RDP classifier against the SILVA (SSU123) 16S rRNA database using a confidence threshold of 70% was used to analyze the taxonomy of each 16S rRNA gene sequence.

### 2.9 Statistical analysis

Data are presented as mean ± SD. Student's *t*‐test was used to determine the statistical differences among two groups, whereas one‐way analysis of variance (anova) was used for comparisons between multiple groups. *p* value less than .05 was considered statistically significant. GraphPad Prism 6.0 was used for analyses. For gut microbiota analyses, Wilcoxon test was used for two independent samples while Kruskal–Wallis test was applied for inter group analysis.

## 3 RESULTS

### 3.1 *L. johnsonii* 6084 reduced the mortality and inflammation in septic mice

**FIGURE 1:**
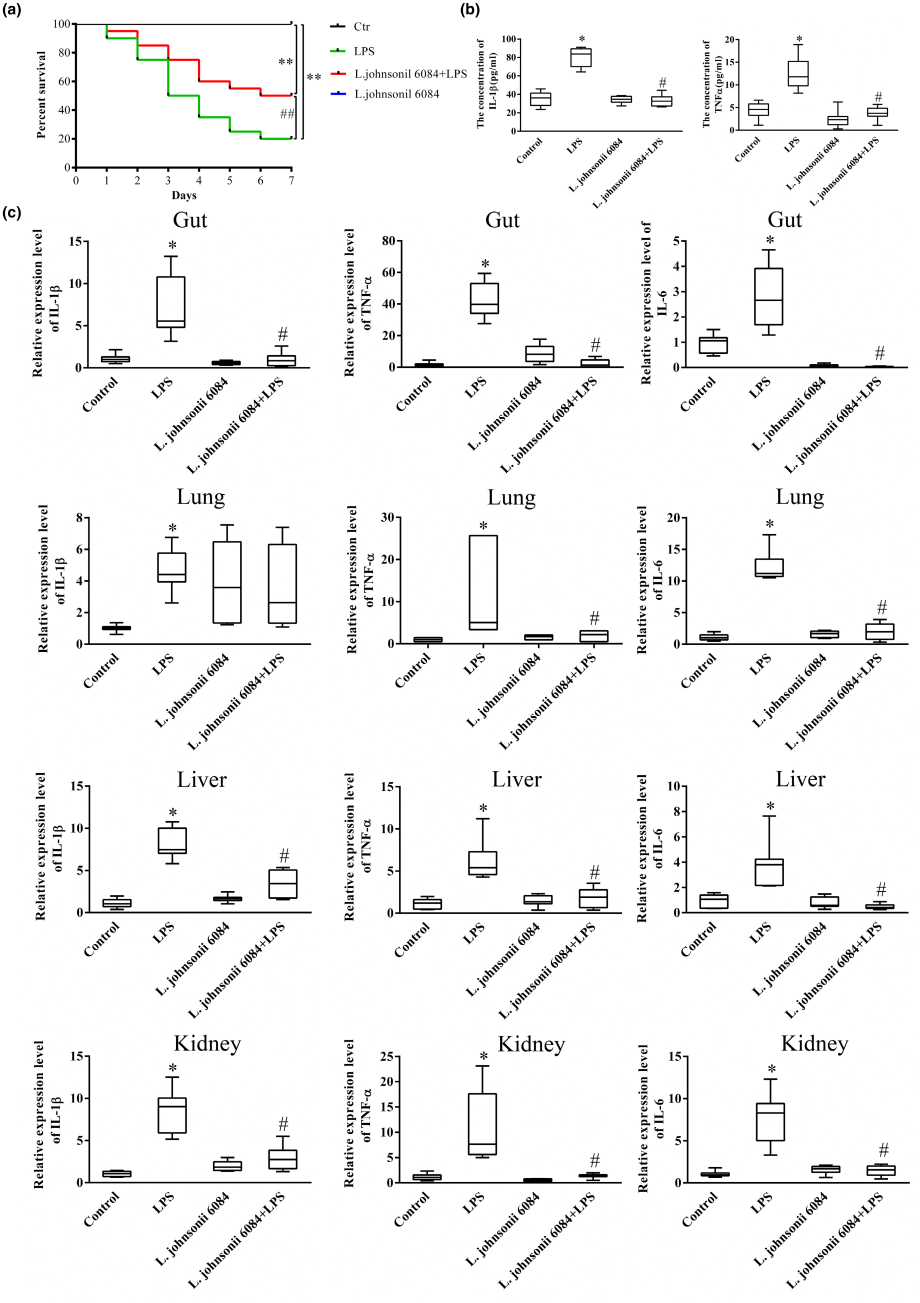
*L. johnsonii* 6084 alleviates the inflammation caused by LPS‐induced sepsis. (a) Survival rates of mice with or without *L. johnsonii* 6084 treatment (*n* = 10). (b) The concentration of IL‐1β and TNF‐α in blood were determined using commercial elisa kits (*n* = 8). (c) *L. johnsonii* 6084 intervention resulted in decreased inflammation of gut, lung, liver, and kidney (*n* = 8). Error bars represent SEM. * *p* < .05 compared with control group. ^#^ *p* < .05 compared with LPS group.

We first evaluated the effect of *L. johnsonii* 6084 on septic mice. Mice subjected to LPS had an approximately 20% 7‐day survival rate, and the survival rate was obviously higher in septic mice treated with *L. johnsonii* 6084 compared with that in the LPS group (50%) (Figure 1a). Consistent with the reduced mortality rate, *L. johnsonii* 6084 treatment significantly decreased the expression of serum inflammatory cytokines, IL‐1β, and TNF‐α, in septic mice (Figure 1b). We further examined the levels of inflammatory cytokines in lungs, livers, kidneys, and intestines. Mice subjected to LPS had a much higher expression of IL‐1β, TNF‐α, and IL‐6 in all tissues while *L. johnsonii* 6084 treatment substantially decreased the inflammatory cytokines (IL‐1β, TNF‐α, and IL‐6) expression in all tissues compared with those in the LPS group (Figure 1c). These results suggested that *L. johnsonii* 6084 treatment could reduce the mortality and inflammation in LPS‐induced septic mice.

### 3.2 *L. johnsonii* 6084 treatment protected against the organ injuries in septic mice

**FIGURE 2:**
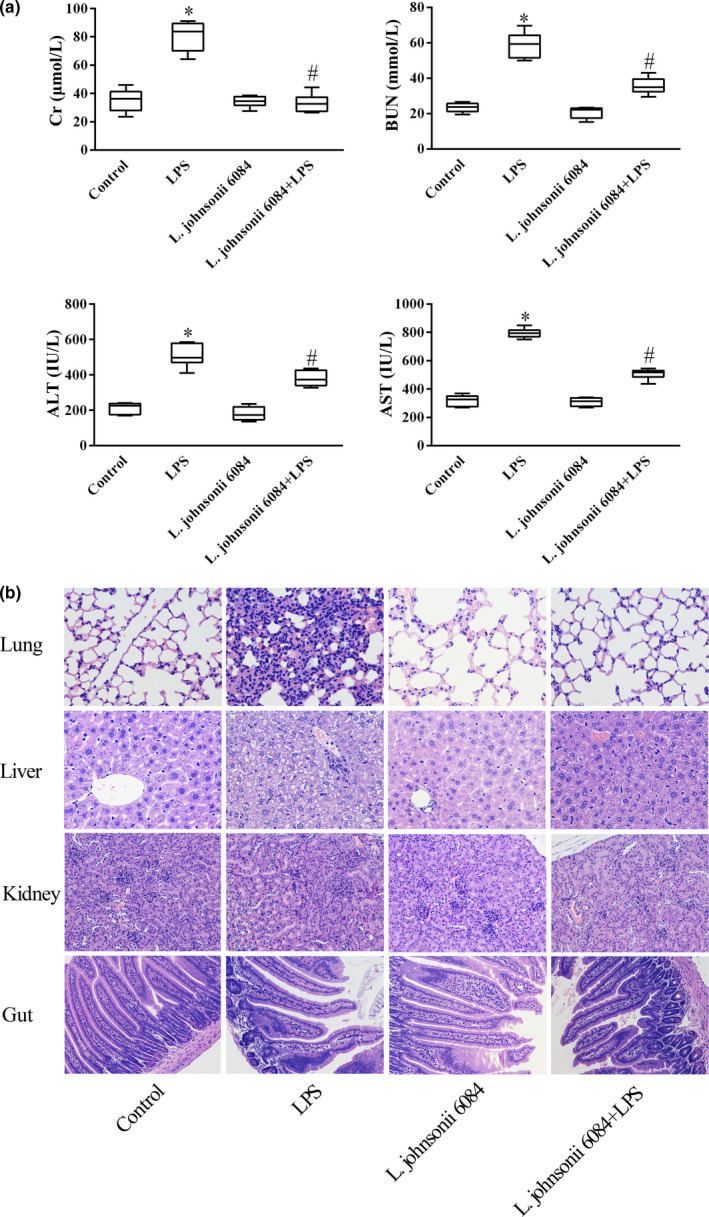
*L. johnsonii* 6084 protected against organ injuries caused by sepsis. (a) *L. johnsonii* 6084 interference resulted in decreased Cr, BUN, ALT, and AST (*n* = 8). (b) H&E staining of lung, liver, gut, and kidney tissues from different groups. Sections were examined and photographed under a microscope (*n* = 8). * *p* < .05 compared with control group. ^#^ *p* < .05 compared with LPS group.

We further verified the effect of *L. johnsonii* 6084 on organ injuries induced by sepsis. Mice subjected to LPS had a much higher levels of Cr, BUN, ALT, and AST than those in control group while *L. johnsonii* 6084 treatment substantially decreased the levels of Cr, BUN, ALT, and AST compared with those in LPS group (Figure 2a). Moreover, H&E staining revealed that organ injuries induced by sepsis were mitigated with *L. johnsonii* 6084 treatment. As shown in Figure 2b, in pulmonary sections, the lung tissue from the septic mice treated with *L. johnsonii* 6084 showed remarkable decreased infiltration of inflammatory cells, alleviated edema and hemorrhage, and less damage of alveolar structures compared with those in LPS group. In liver sections, the liver section from the septic mice treated with *L. johnsonii* 6084 had relieved congestion of veins as well as hepatocyte necrosis compared with those in the LPS group. In the kidney section from the septic mice treated with *L. johnsonii* 6084, there were less necrotic glomeruli and the tubule structure was nearly normal compared with those in the LPS group. In the intestines section from the septic mice treated with *L. johnsonii* 6084, alleviated shortened intestinal villi and less infiltration of inflammatory cells were observed compared with those in the LPS group. These results evidenced that *L. johnsonii* 6084 treatment protected against organ injuries in LPS‐induced septic mice.

### 3.3 *L. johnsonii* 6084 treatment increased microbial diversity and richness in LPS‐induced septic mice

**FIGURE 3:**
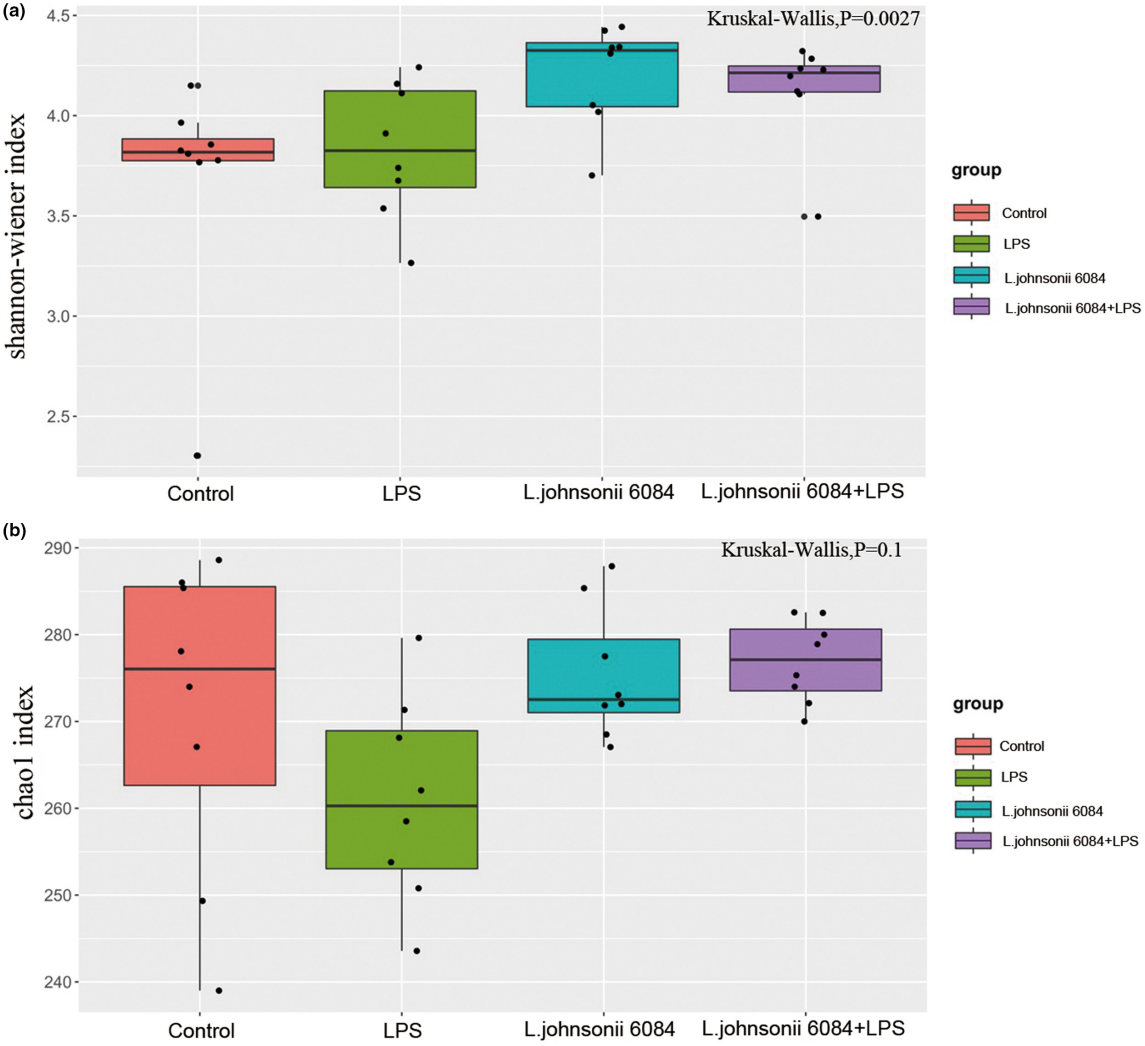
LPS induces significant impact on microbiota composition. (a) Shannon index. (b) Chao1 index. (*n* = 8/group)

We performed 16S rDNA gene amplicon sequencing to detect change of the community structure of gut microbiota. We found that Shannon index (represent the diversity of gut microbiota) and Chaos 1 index (represent the richness of gut microbiota) were significantly reduced in septic mice. With the treatment of *L. johnsonii* 6084, both the indexes increased in septic mice, which reached a level closer to that of the control group. These results suggested that *L. johnsonii* 6084 treatment increased microbial diversity and richness in LPS‐induced septic mice Figure 3.

### 3.4 Administration of *L. johnsonii* 6084 altered the intestinal microbiota composition

**FIGURE 4:**
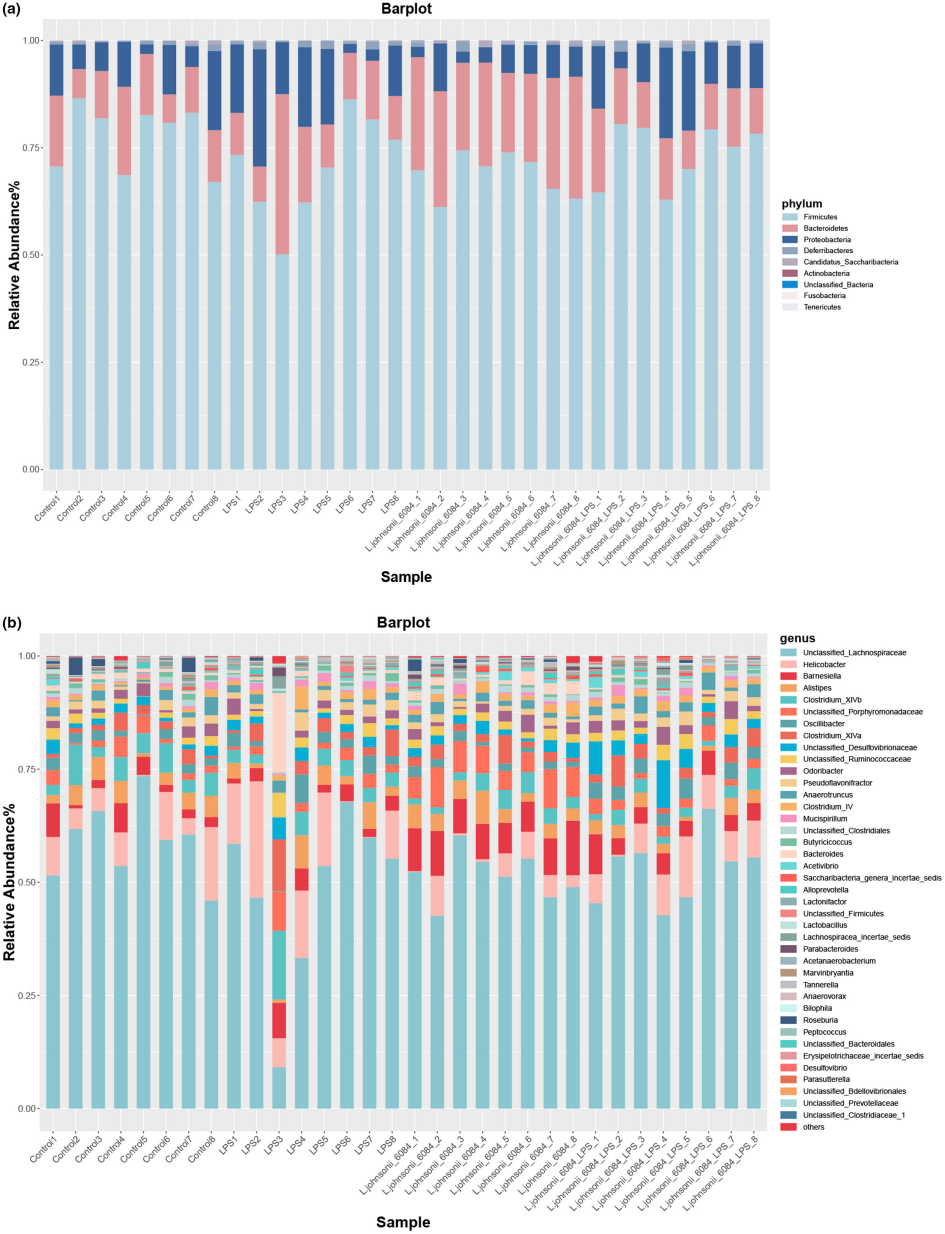
The relative abundances of gut microbiota at family level (a) and genus (b) levels in control, LPS, *L. johnsonii* 6084, and *L. johnsonii* 6084 + septic mice

Nine phyla were detected in microbial profiles of experimental mouse (Figure 4a), among which *Firmicutes*, *Proteobacteria*, *Bacteroidetes*, and *Deferribacteres* were the four major phyla in septic mice, whereas, *Firmicutes*, *Bacteroidetes*, *Proteobacteria*, and *Deferribacteres* were the four major phyla in the samples of control group mice. *L. johnsonii* 6084 treatment changed the abundance of gut microbiota. The intestines microbiota composition was also analyzed at genus levels (Figure 4b).

**FIGURE 5:**
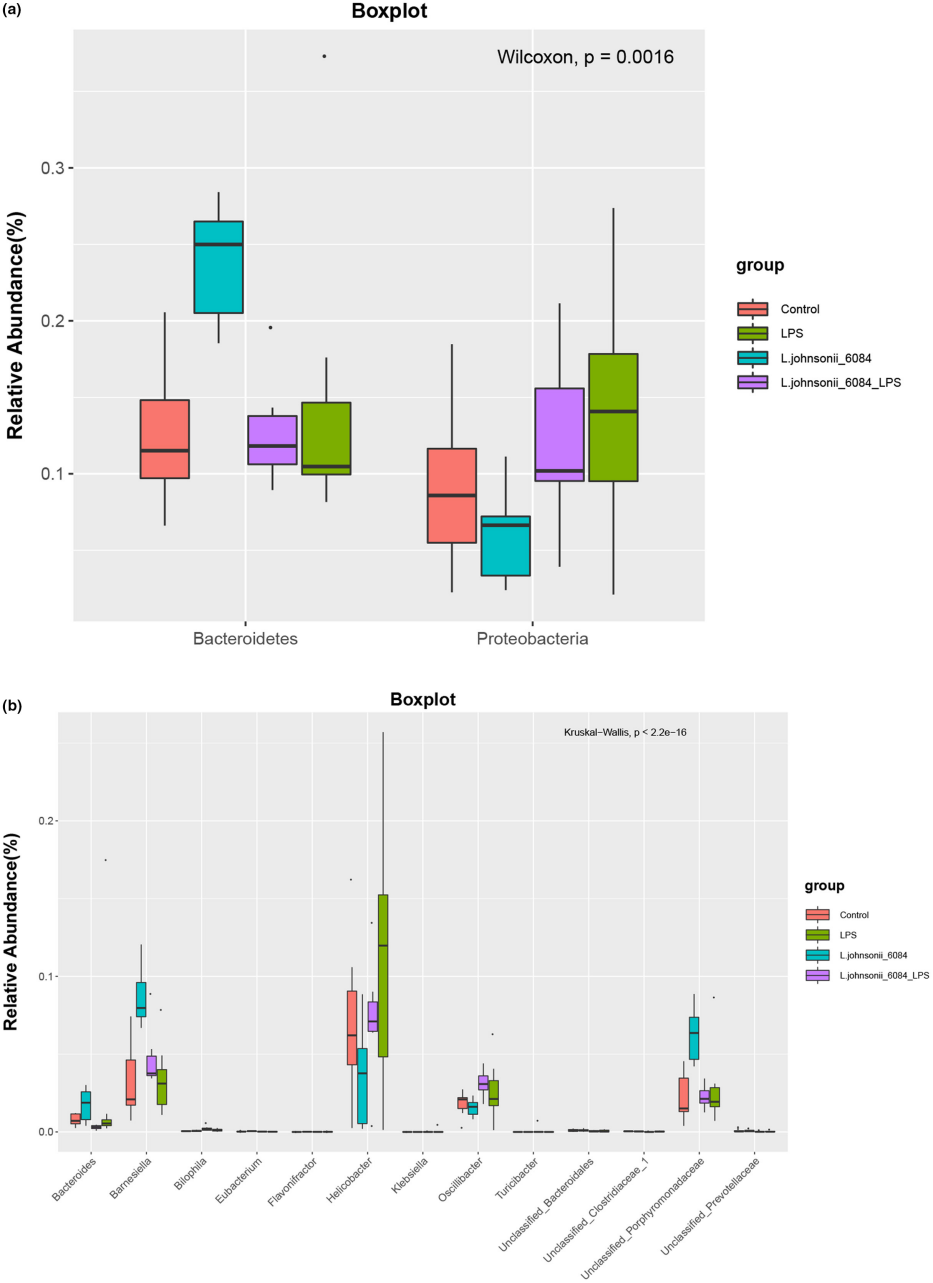
*L. johnsonii* 6084 has a significant impact on microbiota composition (*n* = 8). (a) The change of gut microbiota at phylum level. (b) The change of gut microbiota at genus level.

The differences between gut microbiota of the four groups of mice at the family and genus levels are presented in Figure 5. The relative abundance of *Bacteroidetes* and *Proteobacteria* was significantly different in four groups among all identified phylum (*p* = .0016). *Bacteroidetes* in LPS mouse was significantly increased and *L. johnsonii* 6084 administration decreased dramatically while *Proteobacteria* exhibited an opposite trend (Figure 5a).

Most of the microbiota at a genus level were dominated by unclassified *Lachnospiraceae*, *Helicobacter*, *Barnesiella*, *Alistipes*, and *Clostridium XlVb* while there was two exception LPS‐treated mouse which was dominated by *Bacteroides*. The relative abundance of *Bacteroides*, *Barnesiella*, *Bilophila*, *Eubacterium*, *Flavonifractor*, *Helicobacter*, *Klebsiella*, *Oscillibacter*, *Turicibacter*, unclassified *Bacteroidales*, unclassified *Clostridiaceae*, unclassified *Porphyromonadaceae*, and unclassified *Prevotellaceae* were markedly different in four groups at genera level. *Bacteroides*, *Barnesiella*, *Eubacterium*, *Flavonifractor*, *Klebsiella*, unclassified *Porphyromonadaceae*, and unclassified *Porphyromonadaceae* significantly increased after LPS treatment and its abundance decreased after *L. johnsonii* 6084 administration while *Helicobacter*, *Oscillibacter*, unclassified *Bacteroidales*, and unclassified *Clostridiaceae* exposed an opposite trend (Figure 5b).

**FIGURE 6:**
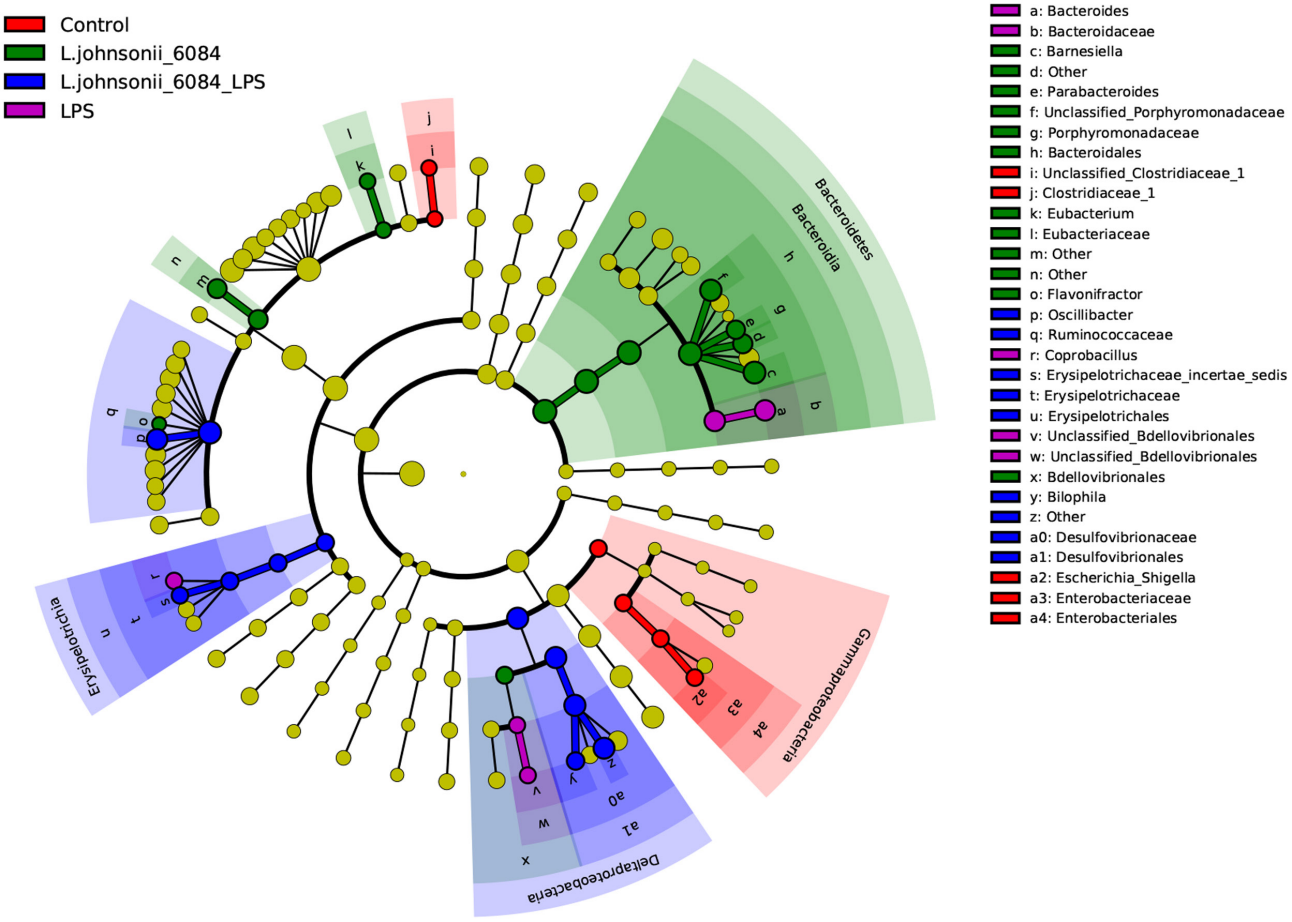
Linear discriminant analysis effect size analyzed the differentially abundant features in different groups. The cladograms represented indicate the bacterial taxa are obviously different among the four groups. This analysis helps to identify a first selection of differential bacterial taxa in the considered groups.

Linear discriminant analysis effect size (LEfSe) was used to determine the differentially abundant features of the four groups which is an algorithm for characterizing genomic features most likely to explain differences between groups. *Bacteroidetes Deltaproteobacteria*, *Gammaproteobacteria*, and *Erysipelotrichia* may be regarded as the key responders of the oral administration of strain 6084 on normal and LPS‐treated mice (Figure 6).

## 4 DISCUSSION

In our study, we investigated the effect of *L. johnsonii* 6084 on the inflammation and organ injury in LPS‐induced septic mice and it was evidenced that *L. johnsonii* 6084 treatment could reduce the mortality and inflammation and protect against the organ injuries in LPS‐induced septic mice, which might result from the alteration of gut microbiota pattern.

Sepsis often causes a systemic inflammatory response. Alleviating patients' inflammatory response is an effective way to relieve the symptoms of sepsis (Bai et al., 2018). Many probiotics can reduce the inflammatory response (Khalique et al., 2019; Yang et al., 2020). Probiotics are microorganisms that colonize the intestinal tract, which can inhibit the growth of harmful bacteria and maintain a stable environment in the intestinal tract. In recent years, it has been found that probiotics have been widely used in clinical practice such as ulcerative colitis, Crohn's disease, intestinal tumors, and other diseases (Jakubczyk et al., 2020). Meanwhile, a large number of clinical and basic studies have confirmed that probiotics have a definite effect on sepsis(Chen et al., 2020; Tsui et al., 2021). Therefore, probiotics are potential candidates for decreasing the symptoms of sepsis. *L. johnsonii* are probiotics that have been used to treat many illnesses. For instance, *L. johnsonii* BS15 can attenuate inflammation in obese mice (Xin et al., 2014); *L. johnsonii* La1 can be useful in preventing bacterial translocation in cirrhosis (Soriano et al., 2012). *L. johnsonii* BS15 intake benefits the neuroinflammation and demyelination in the hippocampus (Xin et al., 2020). Similarly, we observed that *L. johnsonii* decreased the level of inflammatory factors in an LPS‐induced sepsis mouse model.

Various studies reported that the gut microenvironment and the flora composition of sepsis patients were changed significantly (Chen, 2020; Kullberg et al., 2021). During sepsis, the local immune system of the gut was imbalanced, and the gut flora were overproduced, producing a large number of metabolites and toxins, resulting in the damage of intestinal mucosal barrier. The damage of intestinal mucosal barrier will further promote the translocation of intestinal flora and further aggravate sepsis. Previous studies showed shifts in the *Firmicutes* to *Bacteroidetes* ratio of septic mice, as well as reduced microbiota diversity (Ojima et al., 2016; Zaborin et al., 2014). In the present study, the microbiota diversity presented significant differences among groups. Repairing gut microbiota of sepsis provides a new idea for the treatment of it. One of the important ways that probiotics treat diseases is to change the composition of gut microbes (Teixeira et al., 2021; Yin et al., 2021). In this study, the composition of gut microbiota was changed after LPS treatment while it was restored by *L. johnsonii* 6084 treatment especially the relative abundance of *Bacteroides*, *Barnesiella*, *Flavonifractor*, etc. The results indicated that *L. johnsonii* 6084 protects against the organ injuries caused by sepsis through restoring the composition of gut microbiota.

In summary, we demonstrated that *L. johnsonii* 6084 can alleviate inflammation in vitro. It reduced the levels of inflammatory factors caused by sepsis, which may through resistance some pathogenic bacteria enriched in the gut after intraperitoneal injection of LPS promote higher intestinal permeability, and alter the composition of the gut microbiota. *L. johnsonii* 6084 may be used to treat other systemic inflammatory diseases, such as inflammatory bowel disease, systemic inflammatory arthritis, and multiple sclerosis. In our previous study, we found that *S. thermophilus* 19 has the same ability of *L. johnsonii* 6084. Most of the research focused on the therapeutic potential of single probiotics while we can aim to study the efficacy of multiple probiotics in the future. We will investigate the therapeutic potential of the combination of *S. thermophilus* 19 and *L. johnsonii* 6084. Collectively, the results of our study provide a conceptual framework to further text this hypothesis in humans to treat sepsis and other systemic inflammatory diseases.

## CONFLICT OF INTEREST

The authors declare that they have no conflict of interests.

## References

[fsn32989-bib-0001] Adelman, M. W. , Woodworth, M. H. , Langelier, C. , Busch, L. M. , Kempker, J. A. , Kraft, C. S. , & Martin, G. S. (2020). The gut microbiome's role in the development, maintenance, and outcomes of sepsis. Critical Care, 24(1), 278. 10.1186/s13054-020-02989-1 32487252 PMC7266132

[fsn32989-bib-0002] Bai, X. , He, T. , Liu, Y. , Zhang, J. , Li, X. , Shi, J. , … Hu, D. (2018). Acetylation‐dependent regulation of notch signaling in macrophages by SIRT1 affects sepsis development. Frontiers in Immunology, 9, 762. 10.3389/fimmu.2018.00762 29867921 PMC5949384

[fsn32989-bib-0003] Cecconi, M. , Evans, L. , Levy, M. , & Rhodes, A. (2018). Sepsis and septic shock. Lancet, 392(10141), 75–87. 10.1016/S0140-6736(18)30696-2 29937192

[fsn32989-bib-0004] Chen, L. , Li, H. , Chen, Y. , & Yang, Y. (2020). Probiotic Lactobacillus rhamnosus GG reduces mortality of septic mice by modulating gut microbiota composition and metabolic profiles. Nutrition, 78, 110863. 10.1016/j.nut.2020.110863 32593948

[fsn32989-bib-0005] Chen, P. (2020). Gut microbiota and pathogenesis of organ injury introduction. Gut Microbiota and Pathogenesis of Organ Injury, 1238, 1–10. 10.1007/978-981-15-2385-4_1

[fsn32989-bib-0006] Fernando, S. M. , Rochwerg, B. , & Seely, A. J. E. (2018). Clinical implications of the third international consensus definitions for sepsis and septic shock (sepsis‐3). Canadian Medical Association Journal, 190(36), E1058–E1059. 10.1503/cmaj.170149 30201611 PMC6131078

[fsn32989-bib-0007] Ferrer, R. , Martin‐Loeches, I. , Phillips, G. , Osborn, T. M. , Townsend, S. , Dellinger, R. P. , … Levy, M. M. (2014). Empiric antibiotic treatment reduces mortality in severe sepsis and septic shock from the first hour: Results from a guideline‐based performance improvement program. Critical Care Medicine, 42(8), 1749–1755. 10.1097/CCM.0000000000000330 24717459

[fsn32989-bib-0008] Fleischmann, C. , Scherag, A. , Adhikari, N. K. , Hartog, C. S. , Tsaganos, T. , Schlattmann, P. , … International Forum of Acute Care Trialists . (2016). Assessment of global incidence and mortality of hospital‐treated sepsis. Current estimates and limitations. American Journal of Respiratory and Critical Care Medicine, 193(3), 259–272. 10.1164/rccm.201504-0781OC 26414292

[fsn32989-bib-0009] Glenwright, A. J. , Pothula, K. R. , Bhamidimarri, S. P. , Chorev, D. S. , Baslé, A. , Firbank, S. J. , … van den Berg, B. (2017). Structural basis for nutrient acquisition by dominant members of the human gut microbiota. Nature, 541(7637), 407–411. 10.1038/nature20828 28077872 PMC5497811

[fsn32989-bib-0010] Gu, W. J. , Zhang, Z. , & Bakker, J. (2015). Early lactate clearance‐guided therapy in patients with sepsis: A meta‐analysis with trial sequential analysis of randomized controlled trials. Intensive Care Medicine, 41(10), 1862–1863. 10.1007/s00134-015-3955-2 26154408

[fsn32989-bib-0011] Haak, B. W. , Prescott, H. C. , & Wiersinga, W. J. (2018). Therapeutic potential of the gut microbiota in the prevention and treatment of sepsis. Frontiers in Immunology, 9, 2042. 10.3389/fimmu.2018.02042 30250472 PMC6139316

[fsn32989-bib-0012] Han, F. , Wu, G. , Zhang, Y. , Zheng, H. , Han, S. , Li, X. , … Hu, D. (2020). Streptococcus thermophilus attenuates inflammation in septic mice mediated by gut microbiota. Frontiers in Microbiology, 11, 598010. 10.3389/fmicb.2020.598010 33384671 PMC7769777

[fsn32989-bib-0013] Jakubczyk, D. , Leszczynska, K. , & Gorska, S. (2020). The effectiveness of probiotics in the treatment of inflammatory bowel disease (IBD)‐A critical review. Nutrients, 12(7), 1973. 10.3390/nu12071973 PMC7400428 32630805

[fsn32989-bib-0014] Kadri, S. S. , Rhee, C. , Strich, J. R. , Morales, M. K. , Hohmann, S. , Menchaca, J. , … Klompas, M. (2017). Estimating ten‐year trends in septic shock incidence and mortality in United States Academic Medical Centers using clinical data. Chest, 151(2), 278–285. 10.1016/j.chest.2016.07.010 27452768 PMC5310115

[fsn32989-bib-0015] Khalique, A. , Zeng, D. , Wang, H. S. , Qing, X. D. , Zhou, Y. , Xin, J. G. , … Ni, X. (2019). Transcriptome analysis revealed ameliorative effect of probiotic *Lactobacillus johnsonii* BS15 against subclinical necrotic enteritis induced hepatic inflammation in broilers. Microbial Pathogenesis, 132, 201–207. 10.1016/j.micpath.2019.05.011 31077753

[fsn32989-bib-0016] Klaenhammer, T. R. , Azcarate‐Peril, M. A. , Altermann, E. , & Barrangou, R. (2007). Influence of the dairy environment on gene expression and substrate utilization in lactic acid bacteria. The Journal of Nutrition, 137(3 Suppl 2), 748S–750S. 10.1093/jn/137.3.748S 17311971

[fsn32989-bib-0017] Kullberg, R. F. J. , Wiersinga, W. J. , & Haak, B. W. (2021). Gut microbiota and sepsis: From pathogenesis to novel treatments. Current Opinion in Gastroenterology, 37(6), 578–585. 10.1097/MOG.0000000000000781 34419965

[fsn32989-bib-0018] Lahner, E. , Bellisario, C. , Hassan, C. , Zullo, A. , Esposito, G. , & Annibale, B. (2016). Probiotics in the treatment of diverticular disease. A systematic review. Journal of Gastrointestinal and Liver Diseases, 25(1), 79–86. 10.15403/jgld.2014.1121.251.srw 27014757

[fsn32989-bib-0019] Morelli, L. , & Capurso, L. (2012). FAO/WHO guidelines on probiotics: 10 years later. Journal of Clinical Gastroenterology, 46, S1–S2. 10.1097/MCG.0b013e318269fdd5 22955349

[fsn32989-bib-0020] Ojima, M. , Motooka, D. , Shimizu, K. , Gotoh, K. , Shintani, A. , Yoshiya, K. , … Shimazu, T. (2016). Metagenomic analysis reveals dynamic changes of whole gut microbiota in the acute phase of intensive care unit patients. Digestive Diseases and Sciences, 61(6), 1628–1634. 10.1007/s10620-015-4011-3 26715502 PMC4875048

[fsn32989-bib-0021] Paramsothy, S. , Kamm, M. A. , Kaakoush, N. O. , Walsh, A. J. , van den Bogaerde, J. , Samuel, D. , … Borody, T. J. (2017). Multidonor intensive faecal microbiota transplantation for active ulcerative colitis: A randomised placebo‐controlled trial. Lancet, 389(10075), 1218–1228. 10.1016/S0140-6736(17)30182-4 28214091

[fsn32989-bib-0022] Rello, J. , Valenzuela‐Sanchez, F. , Ruiz‐Rodriguez, M. , & Moyano, S. (2017). Sepsis: A review of advances in management. Advances in Therapy, 34(11), 2393–2411. 10.1007/s12325-017-0622-8 29022217 PMC5702377

[fsn32989-bib-0023] Russell, J. A. , Vincent, J. L. , Kjølbye, A. L. , Olsson, H. , Blemings, A. , Spapen, H. , … Grundemar, L. (2017). Selepressin, a novel selective vasopressin V‐1A agonist, is an effective substitute for norepinephrine in a phase IIa randomized, placebo‐controlled trial in septic shock patients. Critical Care, 21, 213. 10.1186/s13054-017-1798-7 28807037 PMC5557574

[fsn32989-bib-0024] Soriano, G. , Sanchez, E. , Guarner, C. , & Schiffrin, E. J. (2012). *Lactobacillus johnsonii* La1 without antioxidants does not decrease bacterial translocation in rats with carbon tetrachloride‐induced cirrhosis. Journal of Hepatology, 57(6), 1395–1396. 10.1016/j.jhep.2012.07.019 22824820

[fsn32989-bib-0025] Teixeira, L. D. , Torrez Lamberti, M. F. , DeBose‐Scarlett, E. , Bahadiroglu, E. , Garrett, T. J. , Gardner, C. L. , … Gonzalez, C. F. (2021). *Lactobacillus johnsonii* N6.2 and blueberry phytophenols affect lipidome and gut microbiota composition of rats under high‐fat diet. Frontiers in Nutrition, 8, 757256. 10.3389/fnut.2021.757256 34722616 PMC8551501

[fsn32989-bib-0026] Tsui, K. C. , Yen, T. L. , Huang, C. J. , & Hong, K. J. (2021). Lactobacillus rhamnosus GG as dietary supplement improved survival from lipopolysaccharides‐induced sepsis in mice. Food Science and Nutrition, 9(12), 6786–6793. 10.1002/fsn3.2630 34925807 PMC8645706

[fsn32989-bib-0027] Wang, H. S. , He, S. H. , Xin, J. E. , Zhang, T. , Sun, N. , Li, L. X. , … Bai, Y. (2021). Psychoactive effects of *Lactobacillus johnsonii* against restraint stress‐induced memory dysfunction in mice through modulating intestinal inflammation and permeability‐a study based on the gut‐brain axis hypothesis. Frontiers in Pharmacology, 12, 662148. 10.3389/fphar.2021.662148 34122081 PMC8189558

[fsn32989-bib-0028] Xin, J. , Zeng, D. , Wang, H. , Ni, X. , Yi, D. , Pan, K. , & Jing, B. (2014). Preventing non‐alcoholic fatty liver disease through *Lactobacillus johnsonii* BS15 by attenuating inflammation and mitochondrial injury and improving gut environment in obese mice. Applied Microbiology and Biotechnology, 98(15), 6817–6829. 10.1007/s00253-014-5752-1 24811405

[fsn32989-bib-0029] Xin, J. , Zeng, D. , Wang, H. , Sun, N. , Khalique, A. , Zhao, Y. , … Ni, X. (2020). *Lactobacillus johnsonii* BS15 improves intestinal environment against fluoride‐induced memory impairment in mice‐a study based on the gut‐brain axis hypothesis. PeerJ, 8, e10125. 10.7717/peerj.10125 33083147 PMC7547597

[fsn32989-bib-0030] Yang, G. Y. , Xia, B. , Su, J. H. , He, T. , Liu, X. , Guo, L. , … Wang, J. F. (2020). Anti‐inflammatory effects of *Lactobacillus johnsonii* L531 in a pig model of Salmonella Infantis infection involves modulation of CCR6(+) T cell responses and ER stress. Veterinary Research, 51(1), 26. 10.1186/s13567-020-00754-4 32093767 PMC7041187

[fsn32989-bib-0031] Yin, J. T. , Sun, W. , Yu, X. Q. , Xiao, X. J. , Li, B. Q. , Tong, Z. H. , … Li, W. (2021). Lacticaseibacillus rhamnosus TR08 alleviated intestinal injury and modulated microbiota dysbiosis in septic mice. BMC Microbiology, 21(1), 249. 10.1186/s12866-021-02317-9 34536996 PMC8449483

[fsn32989-bib-0032] Zaborin, A. , Smith, D. , Garfield, K. , Quensen, J. , Shakhsheer, B. , Kade, M. , … Alverdy, J. C. (2014). Membership and behavior of ultra‐low‐diversity pathogen communities present in the gut of humans during prolonged critical illness. mBio, 5(5), e01361–e01314. 10.1128/mBio.01361-14 25249279 PMC4173762

[fsn32989-bib-0033] Zhang, Y. , Mu, T. , Yang, Y. , Zhang, J. , Ren, F. , & Wu, Z. (2021). *Lactobacillus johnsonii* attenuates citrobacter rodentium‐induced colitis by regulating inflammatory responses and endoplasmic reticulum stress in mice. The Journal of Nutrition, 151(11), 3391–3399. 10.1093/jn/nxab250 34383918

[fsn32989-bib-0034] Zou, Y. J. , Xu, J. J. , Wang, X. , Zhu, Y. H. , Wu, Q. , & Wang, J. F. (2020). *Lactobacillus johnsonii* L531 ameliorates escherichia coli‐induced cell damage via inhibiting NLRP3 inflammasome activity and promoting ATG5/ATG16L1‐mediated autophagy in porcine mammary epithelial cells. Veterinary Sciences, 7(3), 112. 10.3390/vetsci7030112 PMC7558184 32823867

